# Graphene oxide papers with high water adsorption capacity for air dehumidification

**DOI:** 10.1038/s41598-017-09777-y

**Published:** 2017-08-29

**Authors:** Renlong Liu, Tao Gong, Kan Zhang, Changgu Lee

**Affiliations:** 1 School of Mechanical Engineering, Sungkyunkwan University, 2066, Seobu-ro., Jangan-gu, Suwon, Gyeonggi-do 16419 Republic of Korea; 2 Sungkyunkwan Advanced Institute of Nanotechnology, Sungkyunkwan University, 2066, Seobu-ro, Jangan-gu, Suwon, Gyeonggi-do 16419 Republic of Korea

## Abstract

Graphene oxide (GO) has shown a high potential to adsorb and store water molecules due to the oxygen-containing functional groups on its hydrophilic surface. In this study, we characterized the water absorbing properties of graphene oxide in the form of papers. We fabricated three kinds of graphene oxide papers, two with rich oxygen functional groups and one with partial chemical reduction, to vary the oxygen/carbon ratio and found that the paper with high oxygen content has higher moisture adsorption capability. For the GO paper with reduction, the overall moisture absorbance was reduced. However, the absorbance at high humidity was significantly improved due to direct formation of multilayer water vapor in the system, which derived from the weak interaction between the adsorbent and the adsorbate. To demonstrate one application of GO papers as a desiccant, we tested grape fruits with and without GO paper. The fruits with a GO paper exhibited longer-term preservation with delayed mold gathering because of desiccation effect from the paper. Our results suggest that GO will find numerous practical applications as a desiccant and is a promising material for moisture desiccation and food preservation.

## Introduction

Direct dispersion in water of graphene sheets, which have a high specific surface area^1, 2^, is generally considered unattainable^3, 4^, owing to their hydrophobic nature^5^. However, if oxygen functional groups are introduced onto graphene flakes through oxidation or other chemical reaction^6^, water molecules can readily intercalate into the interlayer galleries of the functionalized graphite^7, 8^, suggesting that graphene can be used as a water absorbent^9, 10^. Graphene oxide (GO) is made of a monolayer of graphite oxide and usually gained by oxidative treatments of graphite^1, 11, 12^. While graphene oxide paper is formed, millions of graphene oxide sheets in aqueous solution are assembled into a paper-like material under a directional flow^6^. With a laminated structure, the GO paper has good chemical stability and excellent mechanical stiffness and strength, and can easily be produced in large sizes for macro scale applications^6, 13^. The various aspects of graphene oxide paper have further been exploited, for example, in energy storage, gas sensor, molecular separation, piezoelectricity, supercapacitor, adsorption and so on, aiming to widen its application^7, 9, 14–16^. It is known that functional groups in the GO structure open gaps between the stacked platelets that are approximately 1 nm wide^14, 17^. These gaps in GO paper provide channels for transportation and storage of water molecules. In addition, these hydrophilic groups can adsorb and retain water molecules forming hydrogen bonds. Water molecules are adsorbed physically by being trapped inside capillaries or on the surface, yet their chemical properties remain unchanged^18–20^. Therefore, the moisture absorption process is reversible, thus, it is highly probable that GO papers can be used as a physical absorption desiccant.

Water adsorption in porous material is crucial in the industrial processes and biological systems. One of the monumental work promoting the development of porous silica industry was done by Zhao *et al*. in the 1990s^21^. In their study, well-ordered hexagonal mesoporous silica with uniform pore sizes of 300 Å were first synthesized. Recently, Furukawa *et al*. and Jérôme Canivet *et al*. systematically studied the impressive water uptake performance of porous metal-organic frameworks and related water capture materials^22, 23^.

In this study, we systematically examined the characteristics of moisture adsorption of GO paper with different conditions of surface functionalization. For this, we firstly synthesized three types of GO with controlled oxidation levels: GO from Hummers method (hGO), GO from K_2_S_2_O_8_ enhanced Hummers method (kGO), and reduced GO (rGO). We measured the water adsorption capacity of hGO with varying relative humidity (RH), then repeated with kGO and rGO in order to observe the influence of GO oxidation on adsorption capability and to investigate the mechanism behind the adsorption. Overall, the adsorption capability of kGO was found to be higher than that of hGO and rGO due to the differences in their capillary sizes and the amount of hydrophilic groups. We also observed that the mechanism of water molecule adsorption varied depending on the density of the functional groups. As a practical application, we performed fruit preservation experiment using GO paper and found that the fruit stayed fresh longer using GO than without it. We also tested the adsorption of several organic molecules to demonstrate the feasibility of utilizing GO as an effective physical adsorption desiccant.

## Results and Discussion

### Characterization of different graphene oxide

**Figure 1:**
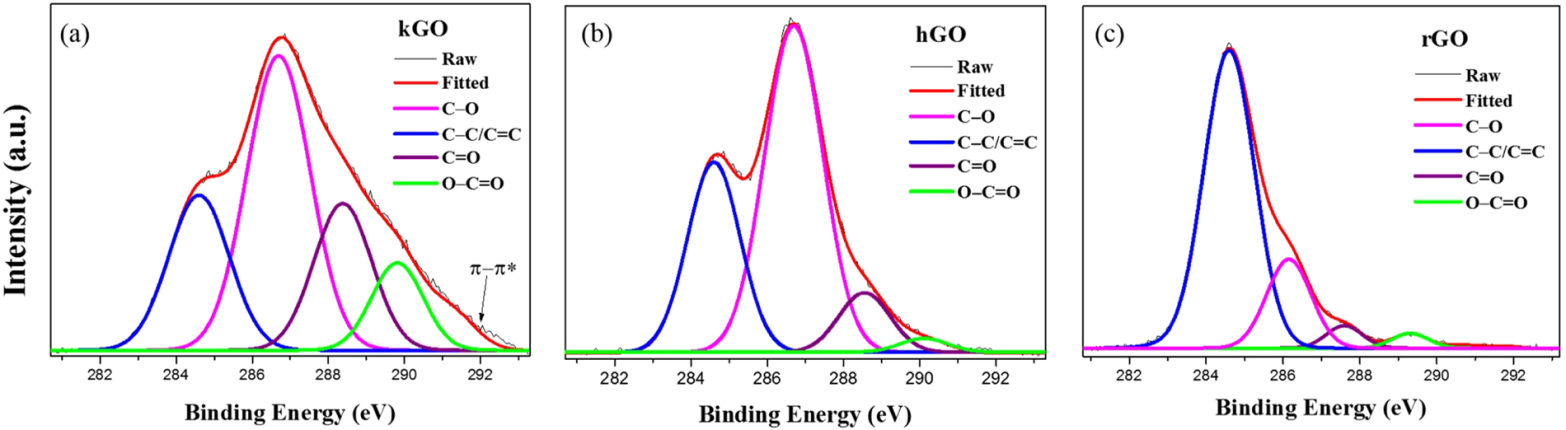
High resolution C-1s XPS spectroscopy for (**a**) hGO, (**b**) kGO and (**c**) rGO membranes.

The effectiveness of an oxidation process of GO is often evaluated by the carbon/oxygen ratios of the graphene oxide. Based on the elemental analysis by X-ray photoelectron spectroscopy (XPS) shown in Fig. 1, the amount of the sp2 carbon atoms in the as-prepared hGO was higher than in the as-prepared kGO. The O:C ratio in kGO was 1:1.5 and 33% higher than in hGO (1: 2). For rGO, the O:C ratio decreased to 1:9.1 after 2 hours of thermal reduction of hGO in air at 200 °C. Figure 1 also presents the typical C1s spectra of the sample surface. Hydroxyl/epoxy group content were comparable with each other. However, a relatively small amount of the carboxyl groups were identified in both spectra of graphene oxides. The relative concentrations of C–O, C = O and C(O)O bonding in hGO were 58%, 9% and 2% respectively. In kGO, the concentrations of C–O, C = O and C(O)O bonding were 41%, 22% and 12%, which indicates a significant increase in carbonyl and in carboxyl groups (Fig. 1a,b)^24, 25^. In contrast, rGO fabricated from this low temperature thermal reduction process contained far less oxygen-containing functional groups (Fig. 1c)^26^.

### Moisture adsorption characteristics of graphene oxide papers

We varied the relative humidity from 0 to 98% in order to investigate the change in adsorption capacity under different humidity conditions. The adsorption capacities were represented by water uptake per unit weight of absorbent GO paper and with unit g/100 g. This, however, is a dynamic equilibrium and changes with relative humidity and temperature.

The water uptake (M) of GO is calculated as:

1 $$M=\frac{m-{m}_{0d}}{{m}_{0d}}$$

where m is the mass of the GO (with moisture) and m_od_ is the oven-dry mass of GO (i.e. no moisture).

**Figure 2:**
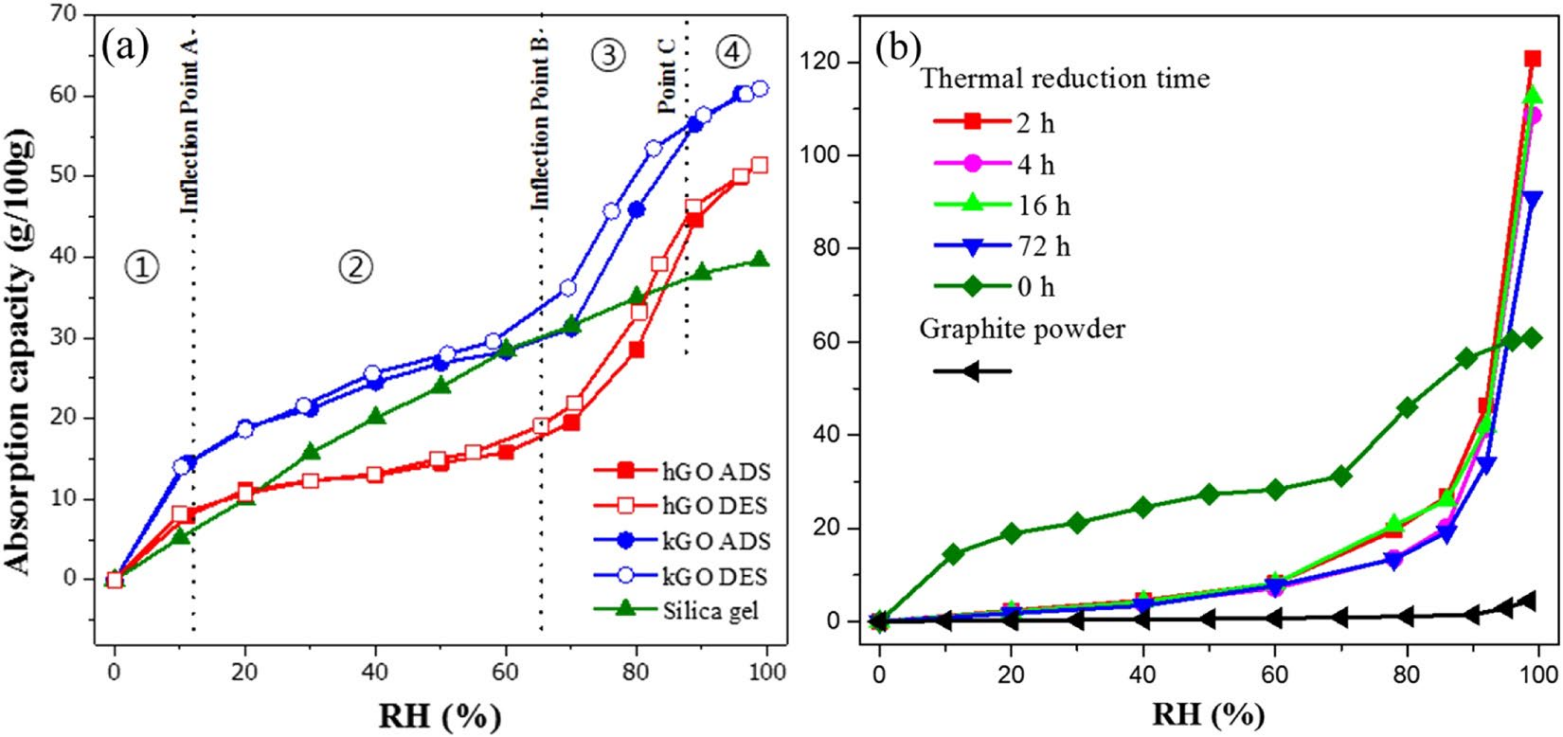
Adsorption capacity for different GO papers. (**a**) Adsorption/desorption isotherm (H_2_O) of 3 kinds of adsorbents. Filled and open symbols represent adsorption and desorption branches respectively. ① First step at low partial pressure region. ② Flat region. ③ Second step at high partial pressure region. ④ Saturation region. (**b**) Adsorption isotherm (H_2_O) of graphite powder and 200 °C thermal reduced hGO, varied by different reduction times.

Generally, adsorption increases with the concentration in the adjacent fluid^27, 28^. As such, water adsorption capability of GO membrane increased with humidity as shown in in Fig. 2(a). The water adsorption capacities of kGO were found to be higher than that of hGO and silica gel, which is a reference material for moisture adsorption test, in all humidity conditions. The maximum adsorption capacities of kGO, hGO and silica gel were 60.9 g/100 g, 51.4 g/100 g and 39.6 g/100 g respectively, which occurred at 98% RH for all tested materials.

The sorption isotherm of kGO and hGO is consistent with Type IV adsorption isotherm which describes monolayer and multilayer adsorption and capillary condensation^29, 30^. These cases are always associated with porous solids in which the adsorbates condense in the small pores at a pressure below saturated vapor pressure. Based on this theory, we can speculate that unlike silica gel, the adsorption capacity of the two types of GO sharply increase at low partial pressure regions of water vapor because of the strong interaction between GO and water molecules^31^. The layer number of water molecules intercalating into the interlayer galleries of GO paper can be estimated based on X-ray diffraction (XRD) data under various humidity condition as shown in Table S1. At low humidity, water molecules tend to be adsorbed onto the surface of the GO membranes first and then penetrate into the GO laminates, forming a monolayer of water on the surface of GO platelets^32–34^. Intermediate flat region ② and inflection point A in the isotherm correspond to the formation of the water monolayer. By increasing the humidity further, multilayer adsorption of water molecules begins. Inflection point B indicates the completion of the monolayer and the onset of multilayer adsorption. At higher relative vapor pressure (region ③), a significant increase in uptake of water occurs for GO membranes, followed by the second step, which appears at a high relative humidity approaching 98% RH before reaching the saturation level. This is generally explained on the basis of a possibility of water molecule condensation in capillary pores of meso-porous materials. Additionally, a hysteresis loop is associated with the secondary pore filling process of capillary condensation^30^. However, the graphene oxide membrane is well known as a kind of nano-meter porous material, and its water adsorption model will be further discussed below with X-ray diffraction data^17^. Eventually, when multilayer of water molecules forms at inflection point C, the GO membrane reaches its saturation state in region ④.

Considering the functional group dependence of water adsorption in the cases of hGO and kGO, it is expected that the water adsorption capacity in rGO would be lower due to the fewer number of functional groups on the surface of the flakes. In Fig. 2(b), we notice that rGO has poor absorbability at relatively low humidity, but shows much better absorbability of water at high humidity (from 95% RH). This is identical to Type III adsorption isotherm, which shows solid absorbent bearing characteristics of weak gas-solid interaction^35^. The weak interaction between water molecules and rGO surface leads to the small uptake at low relative vapor pressure because of significantly reduced functional groups. However, once a water molecule has become adsorbed, the water-water hydrogen bond promotes the adsorption of further molecules, and the resulting isotherm becomes convex to the pressure axis. Thus, rGO shows a different water adsorption tendency from GO. The characteristic of water adsorption of graphite is somewhat similar with rGO in quality although quantitatively there is a big difference. Graphite, bearing no functional groups with smaller layer interspace is found to maintain weak interaction with water vapor. Every 100 g in the tested graphite powder can only adsorb no more than 1 g of water vapor under the relative humidity of 80%. In spite of a sharp increase of adsorption above 90% RH, the maximum absorbability of graphite is 4.4 g/100 g, which is much lower than rGO. This is due to lack of holes, defects, functional groups on intrinsic graphite flakes compared with rGO.

**Figure 3:**
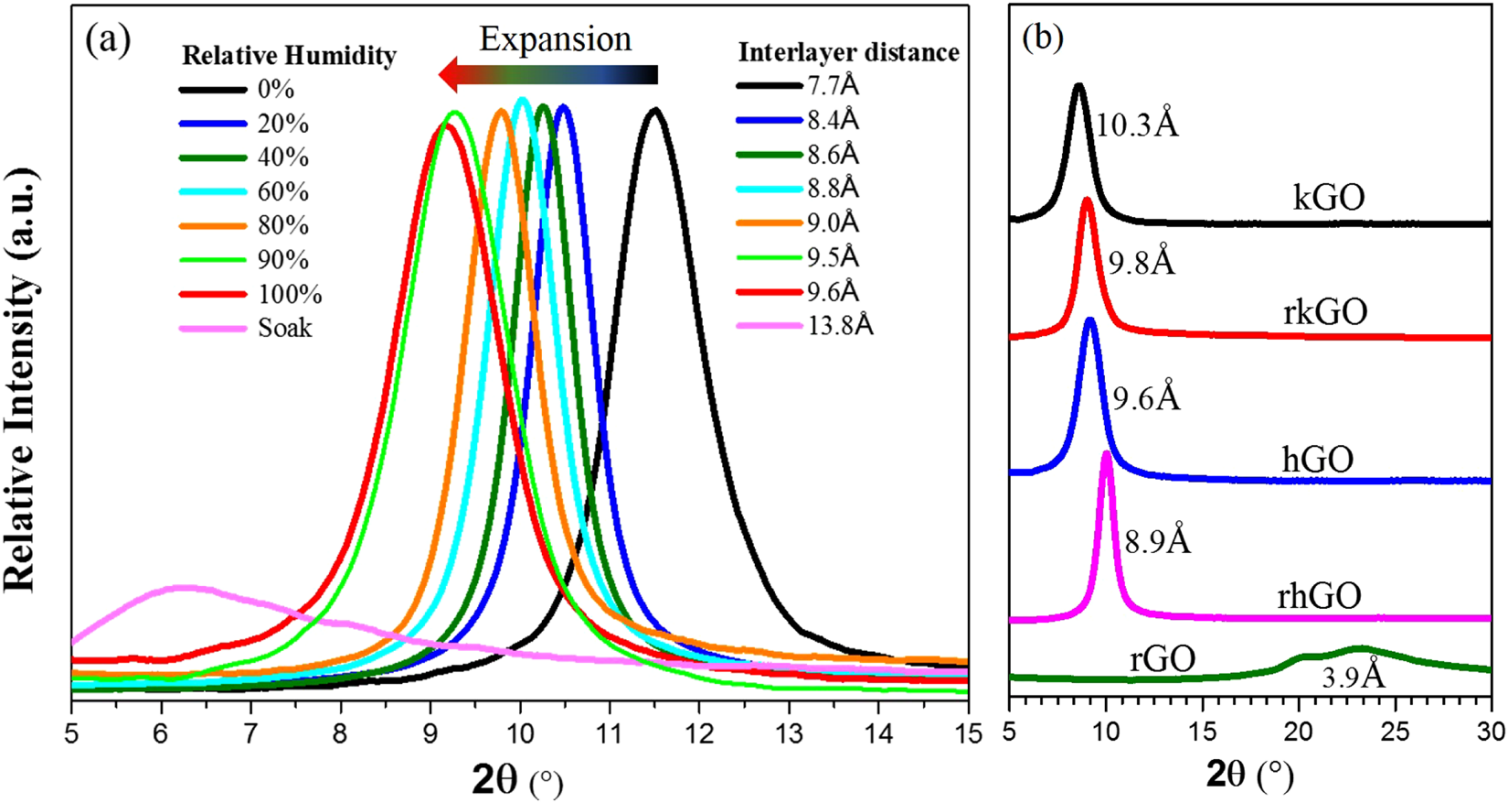
XRD patterns of (**a**) hGO membranes exposed in various RH water vapor, (**b**) all types of GO membranes at 98% RH.

To investigate the water adsorption mechanism in GO, we measured the spacing between the GO flakes under various relative humidity by using XRD. Samples of GO papers were exposed to different vapor pressures of water and examined with XRD at an ambient temperature, as shown in Fig. 3(a). The XRD graphs are for GO papers with 5 µm thickness exposed to water vapor for 12 hours, and the ‘Soak’ graph is for GO soaked in liquid water and is for comparison with other GO samples exposed to water vapor. The sharp peek of (001) reflection indicates that the interlayer distance of every two adjacent graphene flakes is uniform. Exposing the GO samples to water vapor results in a shift in the (001) reflection, which corresponds to an increase in the interlayer distance. It can also be seen that, as the relative humidity increases, the expansion of the corresponding GO membrane increases. The d-spacing which indicatea interlayer distance was calculated by Bragg’s law: λ = 2dsin (θ) where λ is the wavelength of the X-ray beam (0.154 nm), d is the distance between the adjacent GO flakes, θ is the diffraction angle of (001) peak. The spacing between the GO sheets in 98% RH GO is the largest of the samples, up to 9.6 Å, which is 25% larger than that of the dry GO sample. To systematically investigate the influence of oxidation level on interlayer spacing of various GO papers, kGO and hGO were mildly reduced at 100 °C for 4 hours and changed to reduced kGO (rkGO) and reduced hGO (rhGO) respectively. The Interlayer distance of 5 types of GO at 98%RH and 0%RH are exhibited in Fig. 3(b) and Fig. S1 separately. Since kGO is the most oxidized among the GO samples, it has the largest interlayer spacing which allows water molecules to easily permeate and diffuse along the nano-capillaries. This demonstrates that the increase of hydrophilic functional groups content on GO flakes by means of increasing the oxidation level also enlarge the interlayer space of the GO membrane. When the large amount of functional groups on GO flakes were removed after thermal reduction, the interlayer distance of GO shrank down to 3.8 Å forming rGO, and could no longer provide enough interlayer space for water molecules to intercalate. Therefore rGO cannot be regarded as common micro-porous solids in this case and its water adsorption behavior can be well explained by Type III adsorption isotherm theory.

**Figure 4:**
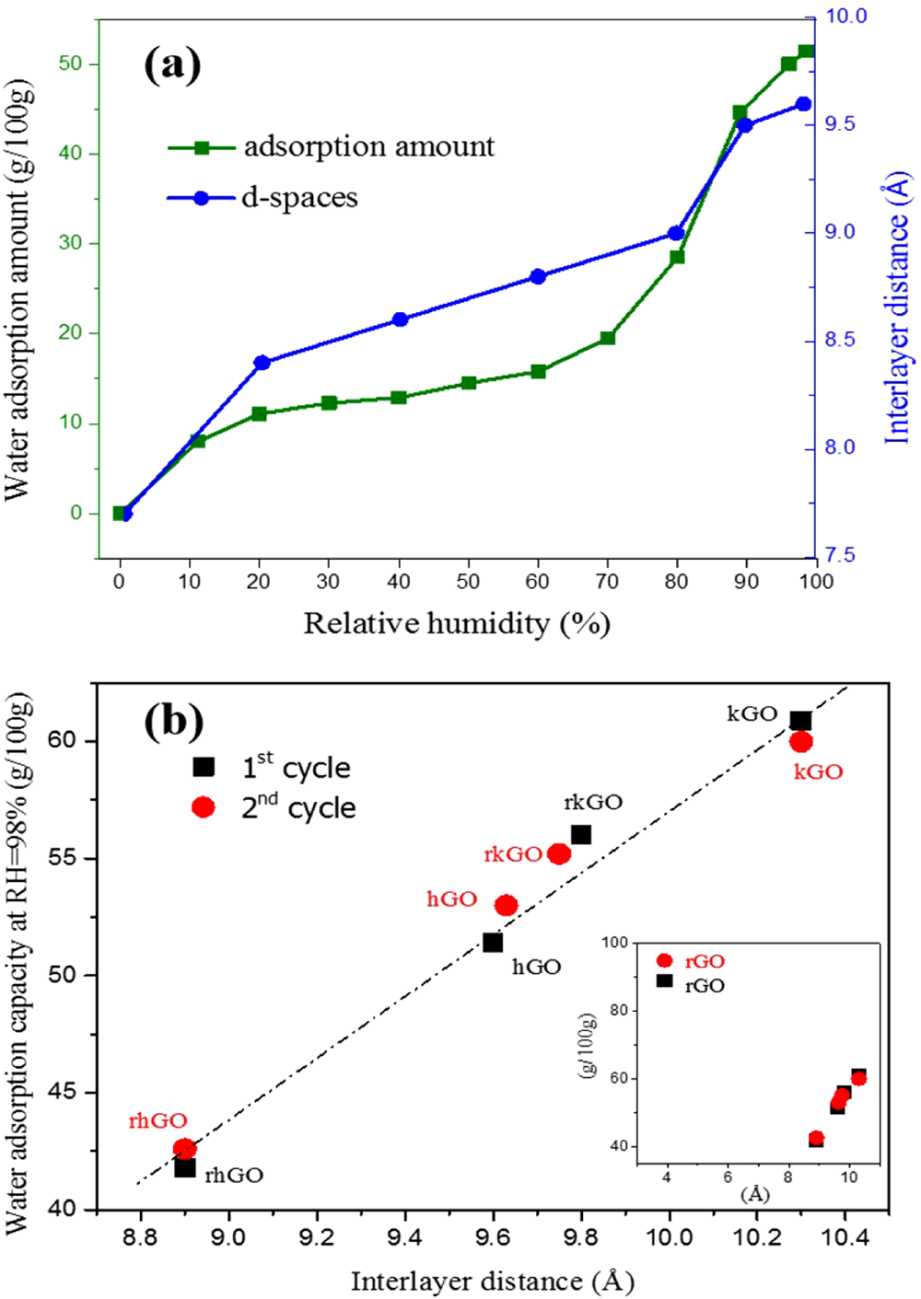
(**a**) Water adsorption capacity vesus the interlayer distance of hGO under different relative humidity, (**b**) Amount of water adsorbed at RH = 98% as a function of interlayer distance for all types of GO membranes; Inlet: water uptake of rGO compared with 4types of GO within 2 cycles when RH = 98%.

The trend of water adsorption isotherm in GO is close to that of GO interlayer distance. In Fig. 4(a), we present the adsorption capacity of hGO together with the as-calculated interlayer distance (d-spacing). Hence, the XRD data helps explain the adsorption behavior of water molecules shown in Fig. 2(a). When the relative humidity increases, the vapor content in the container increases resulting in an increase of the moisture concentration gradient from the environment to GO inner capillaries. Functional groups like hydroxyl and carboxyl groups on the edges of GO flakes begin to adsorb water molecules and help them penetrate through GO inner capillaries. Taking into account the observation that the electron clouds around graphene sheets extend over a distance of 3.5 Å, the initial capillary diameter in dry hGO can be calculated as 7.7 Å − 3.5 Å = 4.2 Å, allowing a monolayer of water (kinetic diameter, 2.68 Å) to easily pass through^36^. Water molecule penetration slows down when one layer of water molecules forms on each GO flake, because the intercalation of water and expansion of GO requires energy to overcome not only the van der Waals force between GO layers but also the hydrogen bond of GO-water interface. When the inter-flake distance is small, van der Waals forces due to deformed ionic clouds around the GO flakes is dominant. However, with the intercalation of more water molecules, the inter-flake distance finally expands over the value of cut-off distance r_c_, and the hydrogen bond formed between intercalated water molecules and their adjacent GO flakes becomes the major interaction forces. Cut-off r_c_ of Lennard-Jones potential is calculated as

2 $${{\rm{r}}}_{{\rm{c}}}=2.5\sigma $$

where σ equals to 0.34 nm for graphite and r_c_ = 0.85 nm^37, 38^.

With the vapor pressure continuously increasing until 70% RH, however, GO internal capillaries expand from 4.2 Å to (9.0 Å–3.5 Å = ) 5.5 Å which provides passages admitting two layers of water molecules abreast (refer to Table S1). Hence, the second layer of water molecules gradually forms in a way similar to the first layer formation of below 20% RH. The moisture adsorption capacity increases sharply simultaneously with the expansion of GO internal capillaries until a bilayer of water molecules forms in the GO capillaries, and the rate of increase begins to slow down at above 90% RH. Even when humidity continues to increase, the interlayer distance of GO reaches a maximum distance of 9.6 Å which is not enough for a third layer of water molecules to intercalate, indicating insufficient driving force for water molecules to expand GO laminates further. At this point, the water capacity of GO reaches saturation. The schematic of water molecule adsorption process in GO stack based on the adsorption isotherm is shown in Fig. S2. The maximum adsorption capacities of kGO, rkGO, hGO and rhGO were 60.9 g/100 g, 56.1 g/100 g, 51.4 g/100 g and 41.8 g/100 g respectively, which occurred at 98% RH.

Since the maximum amount of water adsorbed scales along with the average interlayer distances of the GO samples (Fig. 4b), it is expected that the water molecules adsorbed during the apparent capillary condensation step completely fill the nano-meter interlayer gap. Although the adsorption of water in the GO membrane is generally believed to be the synergistic effect of the surface hydrophilicity and the pore size (interlayer spacing), Fig. 4b shows that the water adsorption capacity of GO is in direct proportion to its interlayer distance, indicating that interlayer distance is the dominant factor in water uptake amount in GO membrane. However, the increase of hydrophilic functional groups content on GO flakes by means of increasing the oxidation level also enlarge the interlayer spaces of GO membranes (Fig. 3b). Thus hydrophilicity can be considered to indirectly influence the water adsorption capacity of GO membrane. On the other hand, this law cannot be applied to rGO. Since the interlayer distance of rGO shrink down to a value smaller than the diameter of water molecule, rGO is not considered to be a micro porous material which can be explained by Type IV adsorption isotherm. In order to address the unique characteristics of absorbent material, water cycling capacity test was also applied. Unlike common absorbent material such as silica, water adsorption capacity of GO samples was maintained stable in 1^st^ and 2^nd^ adsorption cycles, therefore GO membrane desiccant can be repeatedly used and no pollutant is produced during drying of water-saturated GO membrane.

### Water penetration in GO along two directions

**Figure 5:**
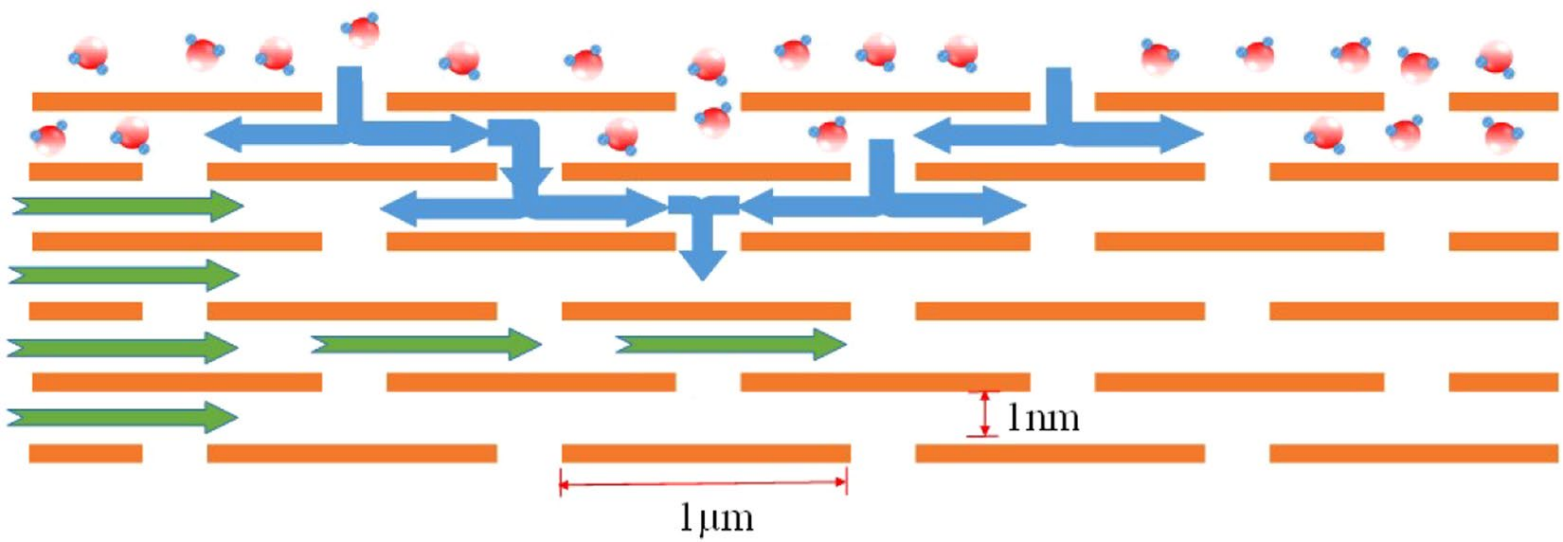
Schematic view for possible penetration through the GO laminates.

To gain insight into water permeation through sub-1-nm-wide capillaries, we employed a model as shown in Fig. 5. The molecules can pass through the capillaries from the top and bottom surfaces or from the edges. Firstly, for the penetration from the top and bottom surfaces, the time to saturate the GO paper exclusively by vertical molecular penetration can be calculated as:

3 $${t}_{1}=\frac{H}{2\times 1nm}\cdot \frac{1\mu m}{2V}=\frac{{10}^{3}\cdot H}{4V}$$

where H is the thickness of the GO membrane, V the diffusion speed of water molecules inside GO, which is assumed to be constant throughout the penetration path in GO. Here we disregard horizontal penetration. The interlayer distance of GO laminates is around 1 nm in high humidity, and the average diameter of GO flakes is tested to be around 1 µm by atomic-force microscopy (AFM) as shown in Fig. S3. The flake sizes distribution of kGO and hGO were can be roughly tested by zeta-size analyzer also as shown in Fig. S4, where the Green curve represents the size distribution of hGO while the red curve represents kGO. It is found that kGO and hGO had similar flakes sizes distribution, so the size factor may not affect the adsorption isotherm too much in this case. Most of these GO flakes have diameters of 1 µm which is consist with our AFM data. Since the vertical penetration involves two opposite directions (up and down), water molecules must flow the distance H/2 to reach the center of the GO membrane. Therefore, it should take a time of 1 µm/2 V for water molecules to saturate each layer of GO in sequence from the surface of the GO membrane to the center.

Secondly, water molecules from the edges of GO paper penetrate into the GO through the horizontal nano-channels as marked with green arrows in Fig. 5. The saturation time regardless of vertical penetration can be easily calculated as

4 $${t}_{2}=\frac{L}{2V}$$

where L is the minimum lateral size of GO paper. In reality, penetration happens from both vertical and lateral directions simultaneously. Yet the resultant velocity of water molecules saturating GO cannot be calculated due to its uncertainty and complexity. However, we can say with certainty that the real saturation time will be close to the smaller value between t_1_ and t_2_. Hence, if the value of H or L increases, the time required for GO saturation also increases.

There should be a critical point when the two direction penetration times are equal, where H/L=2 × 10^−3^. When H/L < 2 × 10^−3^, the vertical penetration dominates. While H/L > 2 × 10^−3^, the horizontal penetration dominates.

**Figure 6:**
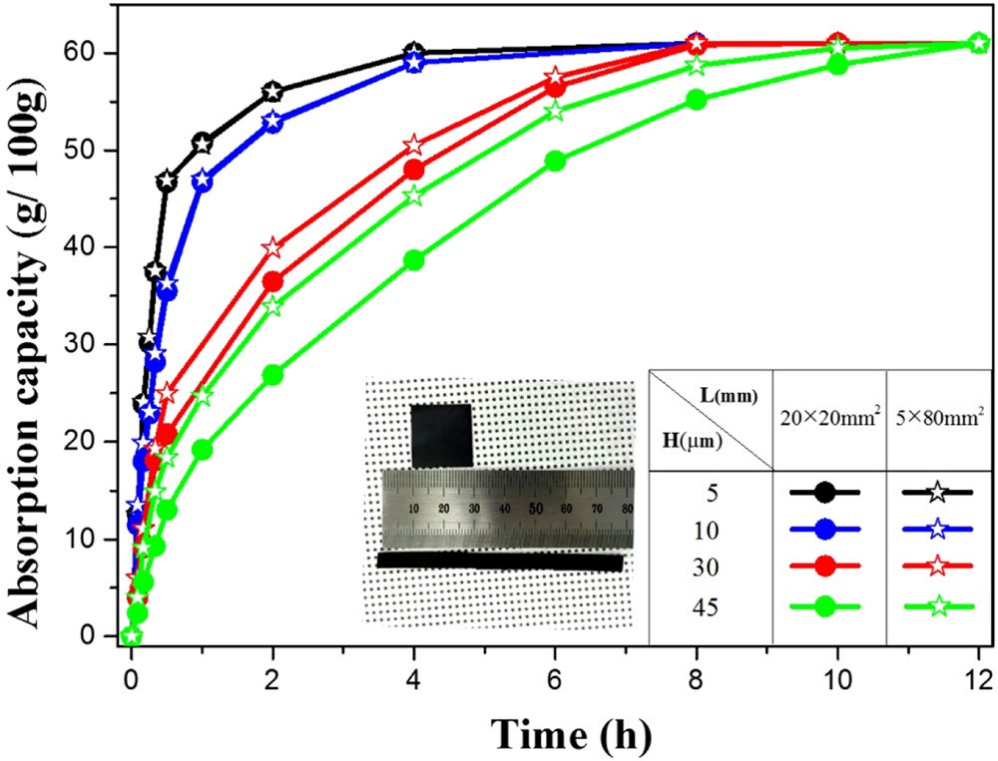
Water adsorption capacity in kGO membranes of different thicknesses and lateral sizes corresponding to time. Inset: two kinds of rectangular GO papers with different lateral sizes (shorter lateral length, L = 5 mm, 20 mm) with the same area (400 mm^2^).

This rough estimation reveals that the vertical direction permeation rate of water molecules is greater and dominant as long as the thickness is roughly less than 20um for 1 cm of lateral size. In order to test the vertical and horizontal direction permeation effects, we fabricated two kinds of rectangular papers with different lateral sizes (length of the short side of the rectangle, L = 5 mm, 20 mm) but with the same area (20 mm × 20 mm). The papers also had 4 different thicknesses (5, 10, 30, 45 um). We measured the permeation rate as shown in Fig. 6. For GO membranes with equal mass and the thickness of 5 µm but with different lateral sizes of 20 mm and 5 mm (see inset of Fig. 6), H/L values are 0.25 × 10^−3^ and 1 × 10^−3^ respectively and are less than 2 × 10^−3^. As predicted, under these conditions, vertical penetration dominates, since the penetration rate does not depend on L.

For 10 µm GO membranes, H/L value of L = 5 mm GO is 2 × 10^−3^ and L = 20 mm GO 0.5 × 10^−3^. Compared with 5 µm GO, the decreased slope of the absorption-time curve indicates a prolonged time of saturation of GO. Here, the permeation rate of the paper with the smaller lateral size is slightly higher, indicating that the horizontal permeation also has some effect, although its path is not the main route of molecular penetration.

In the thicker papers (H = 30 and 45 µm), the H/L values for both L = 5 mm and L = 20 mm GO closely approach and even surpass the critical value of 2 × 10^−3^. The star and circle curves are separated unambiguously. This indicates that horizontal penetration becomes the dominant permeation path of water molecules in these thicknesses rather than vertical permeation.

In order to further evaluate the vapor phase absorption of molecules, we tested absorbability of some solvents such as alcohols and acetone under their corresponding saturated vapor conditions (Fig. S5). The adsorption capacities for these organic molecules were 30–80% lower than that of water vapor. When GO membrane is intercalated with larger molecules, such as ethanol, 1-propanol and acetone, the interlayer distance of GO is expected to be higher. However, this expansion requires energy to overcome the van der Waals force between the GO layers. Larger molecules have to overcome larger energy barrier, which prevents them from penetrating into the GO membrane and lowers the absorption capacity, which agrees with our previous experimental results on liquid phase penetration rates for these solvents^17^. Further investigation on solvents other than water is beyond the scope of this paper and will be performed in the future.

### Application in food preservation

**Figure 7:**
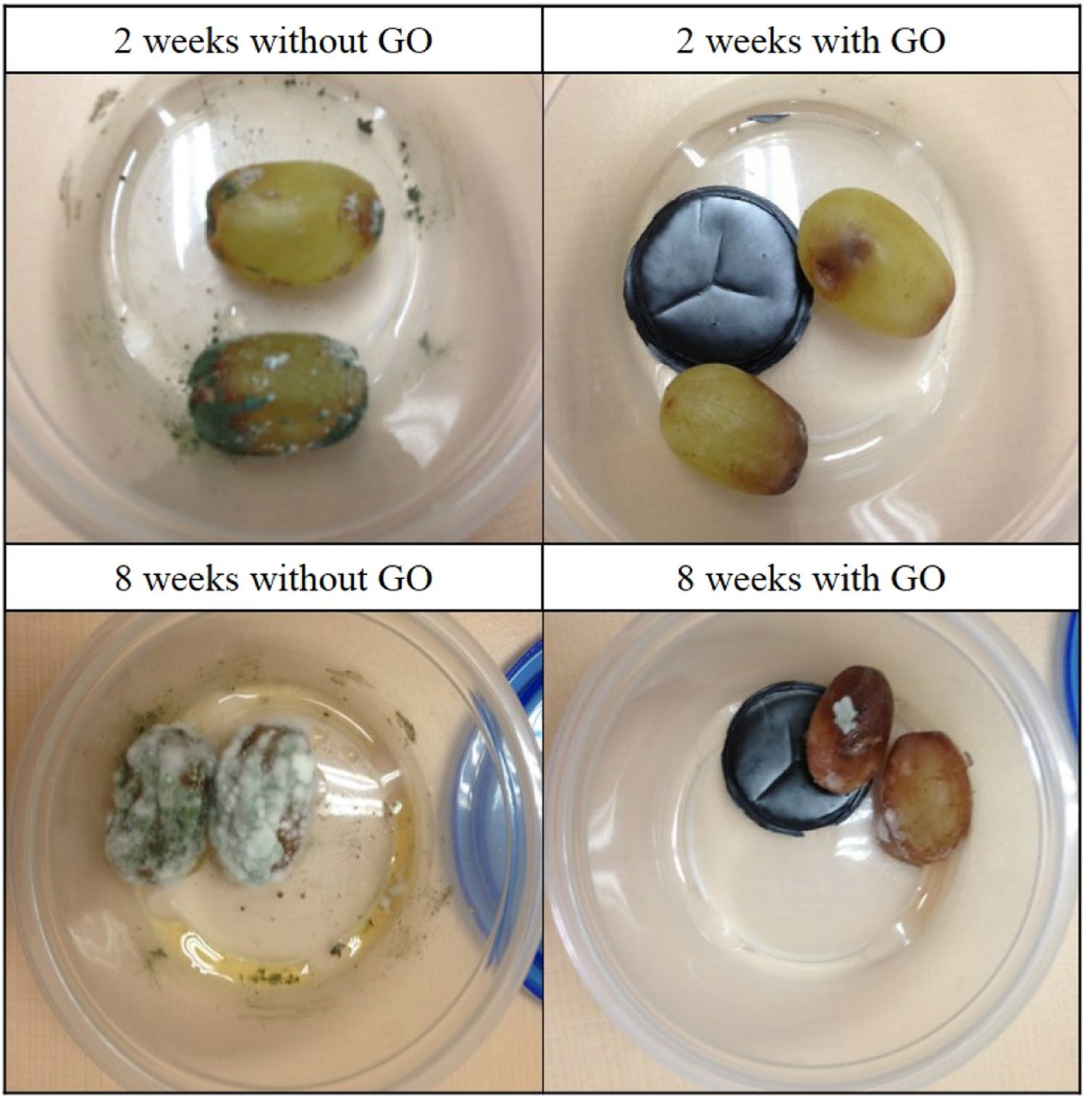
Postharvest grapes preservation test with/without GO after 2 and 8 weeks.

The moisture absorption experiment results clearly reveal that GO paper is an excellent moisture absorber, and we postulated that it could create a dry environment suitable for prolonging food preservation time similar to silica gel. We carried out a preservation test of postharvest grapes as a practical application. As shown in Fig. 7, four grapes were put into two hermetic containers: two in a container with GO paper inside, and the other two in a container without GO paper. An obvious correlation can be readily observed from the size of the fungal population forming on the grapes and the existence of GO. After 2 weeks, dark green mold grew on the surface of grapes and the inner-wall of the hermetic container without GO, while in the container with GO, grapes began to decay only slightly. When observed after 8 weeks, grapes without GO were covered with thick white fungal film and the liquid drop could be observed at the bottom. However, the container with GO was dry, and the decay of grapes was noticeably slower compared to the container without GO. As widely studied, moisture is one of the essential conditions for fungal and bacterial growth. GO was an effective desiccant in controlling the humidity inside the hermetic container by absorbing water. Through a comparison experiment with silica gel, it could be confirmed again that the preservation was due to the moisture reduction. (Fig. S6) Therefore, it is evident that GO has significant potential in inhibiting fungal and bacterial propagation in food by absorbing moisture from the environment. This grape preservation test indicates that GO membranes can be used to extend food preservation time with a simple manufacturing method. A major benefit of using GO paper is that it can easily implemented into a variety of structures like a container or bag by either mixing in a matrix or forming a shape itself to store food or other moisture sensitive materials.

## Conclusion

We characterized the moisture adsorption of graphene oxide papers with different amounts of oxygen-containing functional groups. At low humidity conditions, the more oxidized the paper, the higher adsorption capacity it exhibited. However, at humidity over 60%, the water adsorption capacity of rGO paper increased sharply. In the wide humidity conditions, kGO mostly showed high water moisture absorbability, and the performance was better than the most common desiccant silica gel. Even under the circumstances of extremely low relative humidity, GO can still absorb large amount of water vapor from environment to effectively control the ambient humidity. It undergoes no physical or chemical changes when picking up moisture because GO paper is formed from GO solution with water as solvent. Therefore, after heating to drive the collected moisture exhausted, GO desiccant can be reused. The XRD measurement results reveal that water molecules are adsorbed in monolayer at low humidity up to around 60%, and they begin to accumulate further to form bilayer until saturation. Through adsorption rate tests with GO papers of different thicknesses and sizes, we found the water molecules diffuse into the papers in vertical direction mainly due to the shorter permeation path than horizontal direction. We also carried out a food preservation test with grapes stored in containers since GO showed better moisture adsorption performance than a conventional desiccant, silica gel. It was observed that the grapes with GO paper did not gather mold for more than 2 weeks while those without GO paper gathered mold within 2 weeks. This is an important and direct demonstration that GO paper is an effective desiccant material for food preservation and other various real world applications.

## Methods

### Preparation of graphene oxide papers

hGO was prepared using modified Hummers method from flake graphite (Alfa Aesar, -325 mesh)^39^. 0.5 g of graphite and 0.35 g of NaNO_3_ were placed in a flask followed by adding in 30.7 g of H_2_SO_4_ with stirring in an ice-water bath. 1.95 g of KMnO_4_ was slowly added into the suspension over 1 hr. The mixture was stirred for another 2 hr in the ice-water bath and for 1 day at 35 °C. 50 mL of water was then added while maintaining the temperature at 98 °C. The resultant mixture was further stirred for 1 hr before it was cooled to the ambient temperature, and 1.5 mL of H_2_O_2_ (30 wt % aqueous solution) was slowly added. The mixture was purified with excess aqueous 5 wt % H_2_SO_4_/0.5 wt % H_2_O_2_ solution and followed by di-water for 15 times. The final resultant water solution was passed through a dialysis bag (Sigma Aldrich) for 2 weeks until a pH of ~7 was reached with water as mobile phase to remove the remaining H_2_SO_4_ acid. The hGO colloid suspension was obtained by re-dispersion of pre-dried solid GO in water assisted with ultrasound sonication. For the fabrication of kGO, some modifications has been made to the Hummers method. First, 0.5 g of graphite was placed in a flask followed by addition in 30.7 g of H_2_SO_4_ with stirring in an ice-water bath. 4.5 g K_2_S_2_O_8_ was added into the as-prepared graphite suspension for pre-oxidization. The mixture was stirred for another 2 hr in the ice-water bath and for 3 to 4 days at 35 °C followed by the same procedure as shown in hGO synthesis. All of the graphene oxide papers were prepared by vacuum filtering diluted graphene oxide dispersions through anodisc filter membranes with 0.2 µm pore size followed by a 2 hours vacuum heating process at 60 °C under vacuum of 5 × 10^−2^ Torr.

### Moisture adsorption test

A series of vapor phase moisture adsorption tests were conducted inside an airtight transparent glass container in which a hole was punched at the top for a humidity/air temperature probe to be inserted. Silicone sealant was used to seal the adjoining parts. We changed RH (and, hence, ΔP for water) inside our sealed container by using different concentrations of H_2_SO_4_ solution^40^. To this end, the calibrated concentrated sulfuric acid was diluted with distilled water and placed at the bottom of the container in a small petri dish without any contact with the studied GO membranes, silica gel (Sigma Aldrich, Silica gel Rubin, particle size1-3 mm) or graphite powder (Sigma Aldrich, graphite powder, <20 μm, synthetic). For each concentration of sulfuric acid solution as well as for the distilled water, we measured the water adsorption capacity by the weight increase method. The concentrations we used were conc. 64.8% that provides 10% RH, Conc.58.9% (20% RH), Conc.52.9% (30% RH), Conc.51.0% (40%RH), Conc.43.4% (50%RH), Conc.38.0% (60%RH), Conc.35.0% (70%RH), Conc.26.2% (80%RH), Conc.18.5% (90%RH) and Conc.0% (98.5%RH). Water adsorption tests was taken under atmospheric pressure and room temperature. Each adsorption data was tested for 12 hours to reach water saturation point in GO. The water adsorption capacity in GO was measured by a simple weight increase method.

## Electronic supplementary material

Supplementary information
