# Supplementary material for: Graphene oxide papers with high water adsorption capacity for air dehumidification
Source: Sci Rep. 2017 Aug 29;7:9761. doi: 10.1038/s41598-017-09777-y (PMC5575065; doi:10.1038/s41598-017-09777-y)
Supplement: Supplementary file 1 — Supplementary information [file 41598_2017_9777_MOESM1_ESM.doc]

Supporting Information

**Graphene oxide papers with high water adsorption capacity for air dehumidification**

**Renlong Liu**^1^, **Tao Gong**^2^, **Kan Zhang**^2^, **Changgu Lee**^1,2^*

1. School of Mechanical Engineering, Sungkyunkwan University, 2066, Seobu-ro., Jangan-gu, Suwon,

Gyeonggi, 440-746, Republic of Korea

1. Sungkyunkwan Advanced Institute of Nanotechnology, Sungkyunkwan University, 2066, Seobu-ro, Jangan-gu, Suwon, Gyeonggi, 440-746, Republic of Korea * Corresponding author.

E-mail: peterlee@skku.edu

**Table S1**. Layers of water molecules intercalating into the interlayer galleries of GO under various conditions

| Conditions of GO | d-spacing (Å) | Layer # of water |
| --- | --- | --- |
| RH=0% | 7.7 | 1 |
| RH=20% | 8.4 | 1 |
| RH=40% | 8.6 | 1 |
| RH=60% | 8.8 | 1 |
| RH=80% | 9 | 2 |
| RH=90% | 9.5 | 2 |
| RH=98% | 9.6 | 2 |
| Soaking in water | 13.8 | 3 |
| rGO | 3.8 | 0 |


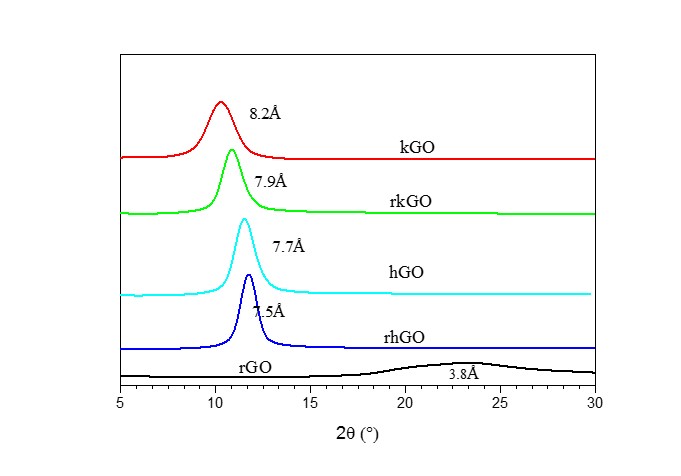


**Figure S1.** XRD patterns of different types of GO membranes at approximate 0% RH.


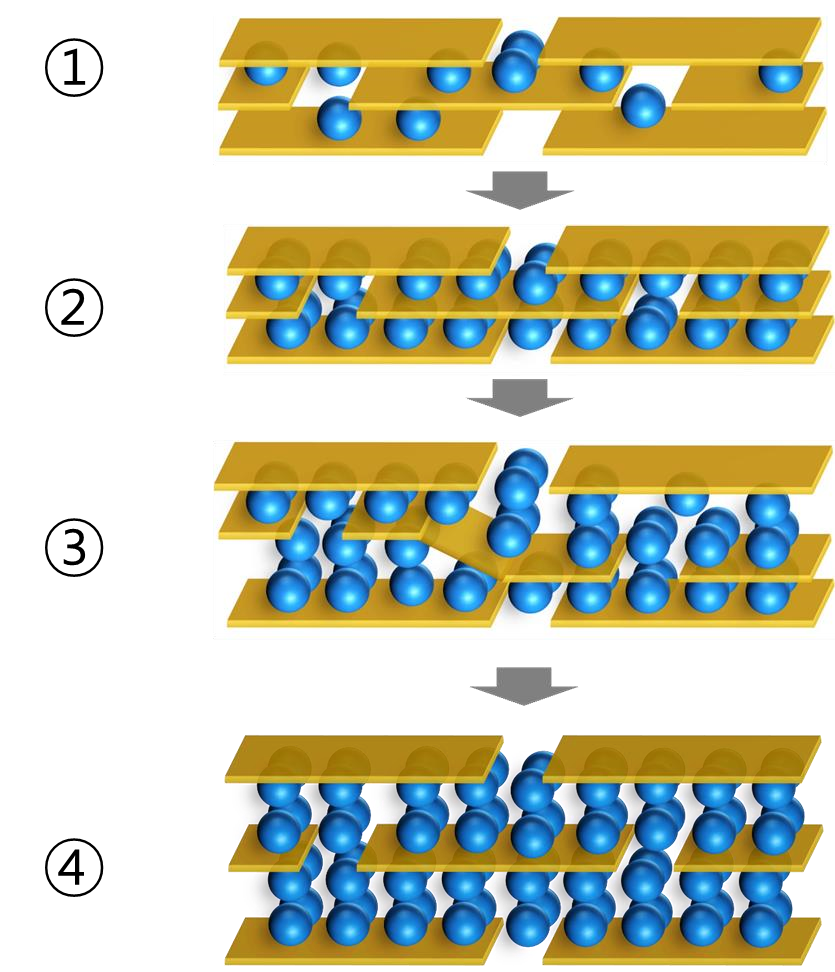


**Figure S2.** Schematic of water molecules adsorption process inside GO flakes corresponding to the adsorption isotherm of GO in figure 2.


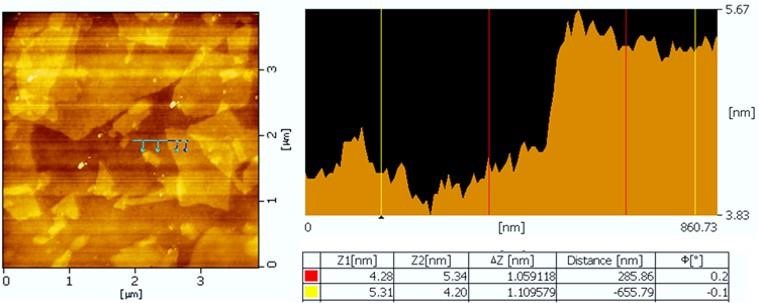


**Figure S3**. AFM study of the nanometer thick of Hummers method graphene oxide (hGO) platelets deposited on the silicon substrate was carried out using an SII Nano Technology Inc.

SPI 4000 in tapping mode. The resulting images shows that the majority of the hGO flakes are around 1 μm and that the thickness of the monolayer is approximately 1 nm


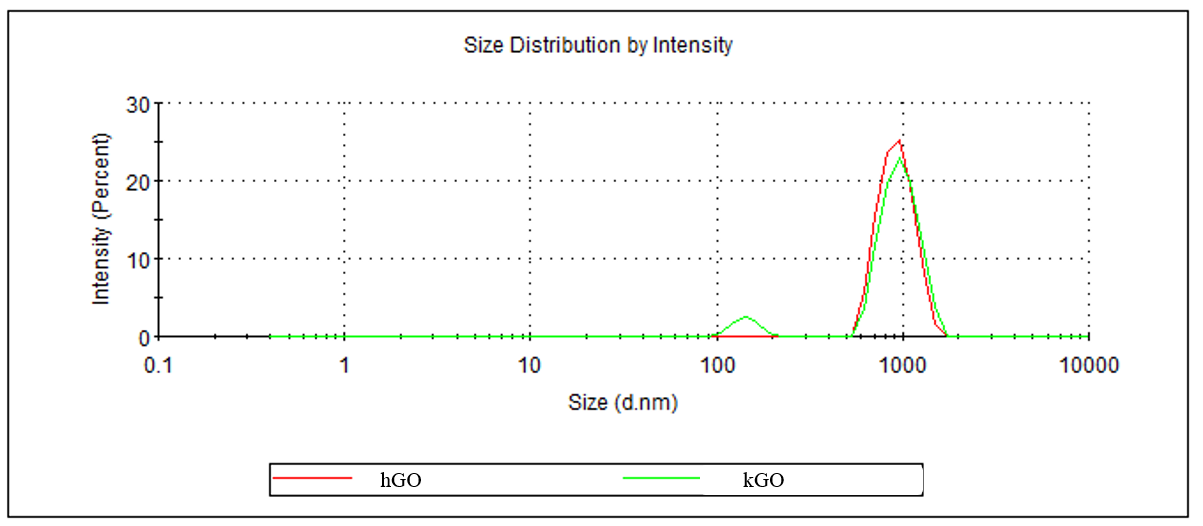


**Figure S4**. Size distribution of kGO and hGO in aqueous solution.


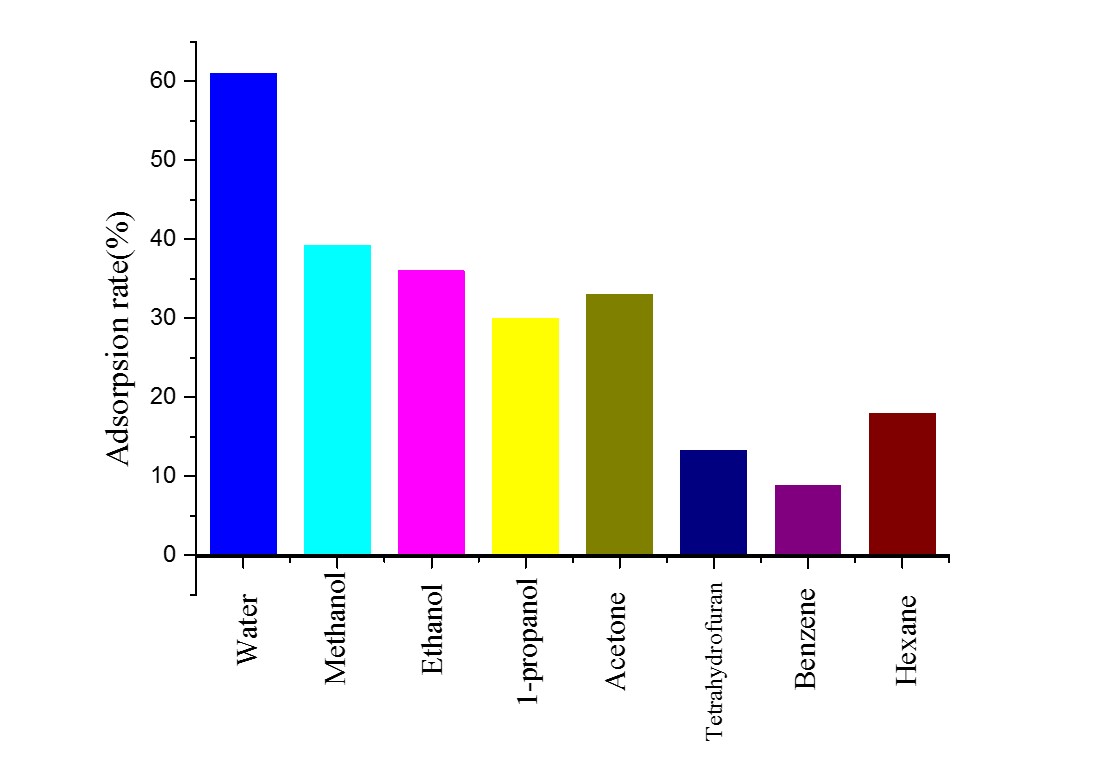


**Figure S5**. GO adsorption of various kinds of volatile organic compounds.

Same batch of postharvest grapes with similar weight were sealed inside 3 same airtight containers at a humidity of 28%RH. There is no desiccants at all in one of the containers, while same uptake capacity of silica gel and kGO were put inside of the other two containers separately. “Same uptake” means the absorption capacity of kGO is 0.35 times higher than that of silica gel (Fig.2) at 28%RH, in order to guarantee that GO and silica gel absorb same amount of moisture, the mass of input kGO is 0.35 times smaller than that of input silica gel. The decay speed of grapes with GO and silica gel were both noticeably slowed down compared to those without desiccants. And the preservative effect of silica gel and GO were equally matched in this case, representing that the moisture absorption property of GO leads to its preservative behavior or at least, it dominates. Note that this experiment is carried out in winter, the decay speed of all grapes were slower than those shown in Fig.7 due to relative low humidity and low temperature.


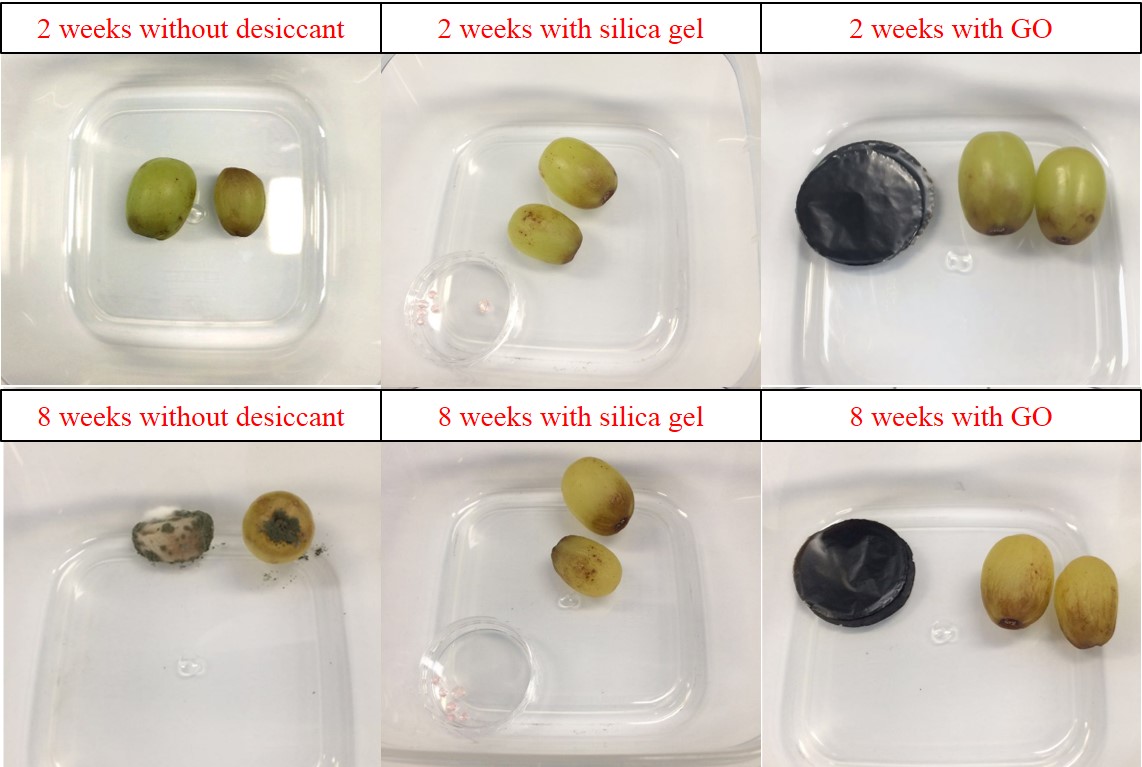


**Figure S6**. Postharvest grapes preservation test with/without GO in comparison with silica gel after 2 and 8 weeks
